# Hypoxia-inducible factor 1 upregulation of both VEGF and ANGPTL4 is required to promote the angiogenic phenotype in uveal melanoma

**DOI:** 10.18632/oncotarget.6868

**Published:** 2016-01-09

**Authors:** Ke Hu, Savalan Babapoor-Farrokhran, Murilo Rodrigues, Monika Deshpande, Brooks Puchner, Fabiana Kashiwabuchi, Syed Junaid Hassan, Laura Asnaghi, James T. Handa, Shannath Merbs, Charles G. Eberhart, Gregg L. Semenza, Silvia Montaner, Akrit Sodhi

**Affiliations:** ^1^ Wilmer Eye Institute, Johns Hopkins School of Medicine, Baltimore, MD, USA; ^2^ The First Affiliated Hospital of Chongqing Medical University, Chongqing, China; ^3^ Department of Pathology, Johns Hopkins University, School of Medicine, Baltimore, MD, USA; ^4^ Departments of Pediatrics, Medicine, Oncology, Radiation Oncology, Biological Chemistry, and Genetic Medicine, Johns Hopkins University School of Medicine, Baltimore, MD, USA; ^5^ Department of Oncology and Diagnostic Sciences, Greenebaum Cancer Center, University of Maryland, Baltimore, MD, USA

**Keywords:** choroidal melanoma, angiogenesis, angiopoietin-like 4 (ANGPTL4), vascular endothelial growth factor (VEGF), hypoxia inducible factor-1α

## Abstract

**Purpose:**

Expression of the hypoxia-inducible factor (HIF)-1-regulated gene product, vascular endothelial growth factor (VEGF), correlates with tumor vascularity in patients with uveal melanoma (UM). While the relationship between HIF-1 and VEGF in cancer is well-studied, their relative contribution to the angiogenic phenotype in UM has not previously been interrogated. Here we evaluate the contribution of HIF-1, VEGF, and a second HIF-1-regulated gene product, angiopoietin-like 4 (ANGPTL4), to angiogenesis in UM.

**Experimental Design:**

UM cells were examined for expression of HIF-1α, VEGF, and ANGPTL4. Their contribution to the angiogenic potential of UM cells was assessed using the endothelial cell tubule formation and directed *in vivo* angiogenesis assays. These results were corroborated in tissue from UM animal models and in tissue from patients with UM.

**Results:**

Inhibition of VEGF partially reduced tubule formation promoted by conditioned medium from UM cells. Inhibition of ANGPTL4, which was highly expressed in hypoxic UM cells, a UM orthotopic transplant model, a UM tumor array, and vitreous samples from UM patients, inhibited the angiogenic potential of UM cells *in vitro* and *in vivo*; this effect was additive to VEGF inhibition.

**Conclusions:**

Targeting both ANGPTL4 and VEGF may be required for the effective inhibition of angiogenesis in UM.

## INTRODUCTION

Uveal melanoma (UM) is the most common adult primary intraocular malignancy [1]. UM arise from melanocytes within the uveal tract (i.e., the iris, ciliary body, and choroid). While iris melanomas are usually benign, ciliary body and choroidal melanomas can be highly metastatic [2]. Metastasis occurs by hematogenous spread and most commonly targets the liver [3]. Despite advances in the last three decades in the diagnosis of - and treatment options for - the primary tumor, we have unfortunately not witnessed a corresponding improvement in patient survival. The detection of hepatic or pulmonary metastatic UM lesions predicts a dismal outcome, with a median survival of only a few months [2].

The recent introduction of gene expression profiling has advanced our understanding of the pathogenesis of UM by identifying two major patient subgroups: class 1 (unlikely to metastasize) and class 2 (very likely to metastasize) [4]. In addition to the prognostic implications of these tests, gene expression profiling has provided researchers new opportunities to explore the complex pathophysiological mechanisms promoting UM growth, progression, and metastatic spread. Ongoing efforts are now focused on identifying the molecular events that help define these two classes, with the ultimate goal of translating these findings to benefit UM patients.

In this regard, the elucidation of the molecular mechanisms governing the transition from a non-angiogenic to an angiogenic phenotype has been central for understanding and treating solid tumors [5]. This is particularly important for tumors that metastasize by hematogenous spread. Intratumoral hypoxia is a driving force for the release of angiogenic stimulators and is independently associated with an increased risk of metastasis and mortality in many human cancers. Hypoxia-inducible factor 1 (HIF-1) is a transcription factor that regulates the expression of secreted factors that mediate the angiogenic phenotype in most cancers [6], and is strongly associated with the class 2 UM gene expression profile [7]. HIF-1 is a heterodimeric protein composed of an exquisitely oxygen-sensitive HIF-1α subunit and a ubiquitous HIF-1β subunit [8]. Under standard tissue culture conditions (20% O_2_), proline residues 402 and 564 on the HIF-1α subunit are hydroxylated by a family of HIF prolyl hydroxylases (PHDs) [9]. Hydroxylated HIF-1α binds to the von Hippel-Lindau (VHL) tumor suppressor protein, which ubiquitinates HIF-1α and targets it for degradation by the proteasome [10, 11]. Inhibitors of the PHDs, including dimethyloxalylglycine (DMOG) and desferrioxamine (DFO), mimic hypoxia by preventing hydroxylation of HIF-1, thereby stabilizing HIF-1α under normal oxygen tension. An additional level of regulation is provided by an asparaginyl hydroxylase, factor inhibiting HIF-1 (FIH-1) [12, 13]. FIH-1 hydroxylates asparagine residue 803 on HIF-1α and prevents binding of the transcriptional co-activator, p300, to HIF-1α, thereby inhibiting its transcriptional activity.

Under hypoxic conditions (<5% O_2_), the ability of PHDs and FIH to hydroxylate HIF-1α is impaired. In the absence of hydroxylation, VHL does not bind to HIF-1α to trigger its degradation, whereas p300 binds to HIF-1α to enhance its transcriptional activity. This results in accumulation of HIF-1α protein, which localizes to the nucleus and binds to HIF-1β forming HIF-1α/β heterodimers, which induce broad changes in gene expression that help adapt the cell, tissue, and organism to low O_2_ conditions. HIF-1 targets include numerous genes that play essential adaptive roles by promoting angiogenesis to increase O_2_ delivery, regulating the metabolic shift from oxidative phosphorylation to glycolysis and lactic acid production to decrease O_2_ demand, protecting cells from acidosis, and influencing adaptive survival mechanisms [14]. These genes work together to collectively promote the survival of cells exposed to hypoxia. HIF-1α protein levels are relatively high in UM cells, even when cultured in 20% oxygen [15]. However, the genes regulated by HIF-1 that mediate the transition from a non-angiogenic to an angiogenic phenotype in UM are not fully known.

Vascular endothelial growth factor (VEGF), a potent angiogenic stimulator, is the best-studied HIF-1-regulated angiogenic gene [16, 17]. Deregulated VEGF expression contributes to the development of solid tumors by promoting angiogenesis, but also by promoting intravasation leading to hematogenous metastasis, which is the major determinant of patient mortality [18]. VEGF levels are elevated in UM tissue, particularly in patients with metastatic disease [19]. While the relationship between HIF-1 and VEGF in cancer is well studied, their relative contribution to the angiogenic phenotype of UM cells has not been previously interrogated. Nonetheless, these laboratory findings have prompted speculation that VEGF inhibition may be a rational approach for the treatment of UM. Enthusiasm has been dampened, however, by disappointing results from clinical trials evaluating therapies targeting VEGF for the treatment of other cancers [20]. These results suggest that tumors express angiogenic factors in addition to VEGF. Here, we examine the relative contribution of HIF-1, VEGF, and another HIF-regulated cytokine, angiopoietin-like 4 (ANGPTL4), to the angiogenic phenotype of UM.

## RESULTS

### HIF-1α expression is increased in UM cells and in tissue from patients with UM

**Figure 1:**
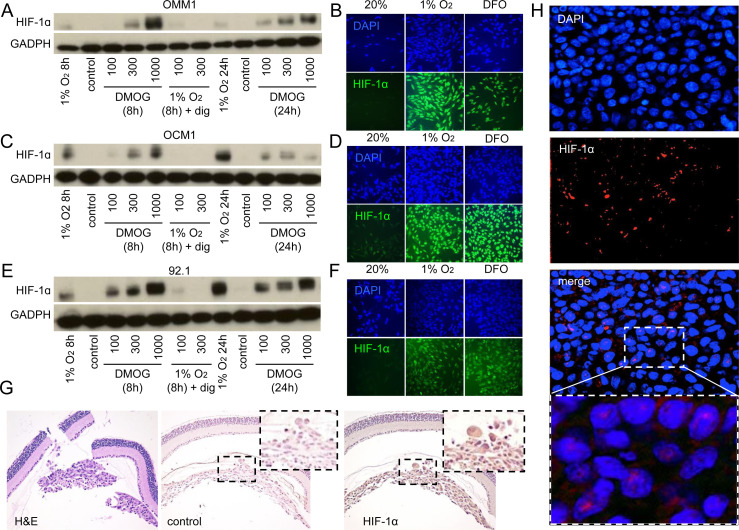
HIF-1α expression is increased in UM cells and in UM patient biopsies **A.**, **C.**, **E.** Immunoblot assays were performed to determine HIF-1α protein levels in UM cell lines (OMM1, OCM1 and 92.1) following exposure to DMOG (300 μM), hypoxia (1% O_2_) or hypoxia and digoxin (dig; 100-300 nM) for 8 or 24 hours and compared to control conditions (20% O_2_). **B.**, **D.**, **F.** Representative images are shown from immunofluorescence analysis of HIF-1α in UM cell lines following exposure to hypoxia (1% O_2_ for 8 or 24 hours) or DFO (100 μM for 8 or 24 hours). **G.** Representative images are shown from immunohistochemical analysis of HIF-1α expression in tumors formed following intravitreal injection of OCM1 cells into mice. Similar results were observed in 3/3 tumors analyzed. **H.** Representative images are shown from immunofluorescence analysis of HIF-1α protein accumulation and nuclear localization in a human UM tumor biopsy. Similar results were observed in 6/6 UM biopsies examined.

Cell lines isolated from UM tissue samples have provided important insight into the molecular pathogenesis of this ocular cancer [22]. To begin studying the role of HIF-1 and the gene products it regulates in the angiogenic phenotype of UM, we examined HIF-1α levels in a well-characterized metastatic UM cell line (OMM1), which was isolated from a subcutaneous metastasis, as well as two primary UM cell lines (OCM1 and 92.1), in the presence of hypoxia (1% O_2_), and observed an increase in HIF-1α protein accumulation and nuclear localization of HIF-1α in all three cell lines (Figure 1A-1F). These results were also observed at 20% O_2_ by treating cells with a prolyl hydroxylase inhibitor (DMOG or DFO). In the presence of hypoxia, accumulation of HIF-1α protein in UM cells was effectively inhibited by co-administration of digoxin (Figure 1A, 1C, and 1E), a well-established inhibitor of hypoxia-induced HIF-1α protein accumulation [23].

To determine the extent that HIF-1α protein accumulated in primary UM tumor cells *in vivo*, we performed orthotopic (intraocular) tumor cell transplantation with OCM1 cells, and observed uniform expression of HIF-1α protein in all tumor cells (Figure 1G). We next evaluated ocular tissue from patients with primary UM. Similar to the results with the UM cells, 6/6 primary UM tissue samples expressed high levels of nuclear HIF-1α (Figure 1H). Collectively, these results support a role for HIF-1α in the molecular pathogenesis of both primary and metastatic UM and further validate the use of these UM cell lines to examine the role of HIF-1α and the genes it regulates in the angiogenic phenotype of UM.

### HIF-1α expression is necessary and sufficient for the angiogenic potential of UM cells

**Figure 2:**
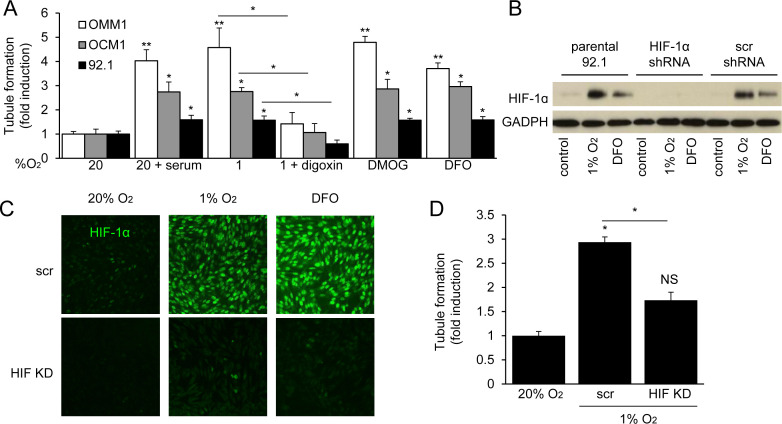
HIF-1α is necessary for the angiogenic potential of UM cells **A.** Formation of tubules by human microvascular endothelial cells (HMECs) treated with conditioned medium from UM cell lines cultured under serum starved conditions (1% FBS) and exposed to 20% O_2_, hypoxia (1% O_2_), hypoxia and 100 nM digoxin, or prolyl hydroxylase inhibitor (300 μM DMOG or 100 μM DFO) in 20% O_2_. Treatment with 10% FBS (serum) is used as a positive control for the tubule formation assay. **B.**, **C.** Expression and nuclear localization of HIF-1α were analyzed by immunoblot **B.** and immunofluorescence **C.** assays in parental 92.1 cells and subclones stably expressing either a short hairpin RNA (shRNA) targeting HIF-1α (HIF KD) or a scrambled control (scr) shRNA. **D.** Aliquots of conditioned medium from parental 92.1 cells exposed to 20% O_2_, or subclones expressing control shRNA (scr) or shRNA targeting HIF-1α (HIF KD) and exposed to 1% O_2_, were incubated with HMECs and the effect on tubule formation was determined. *P < 0.05; **P < 0.01

To assess the relative contribution of hypoxia and HIF-1α to the angiogenic phenotype of UM, we treated cultured human microvascular endothelial cells (HMECs) with conditioned medium and observed a potent induction of tubule formation in the presence of medium conditioned by UM cells exposed to 1% O_2_ (Figure 2A), similar to that observed after treatment with 10% serum, suggesting that secreted factors expressed by hypoxic UM cells promote angiogenesis. To confirm the role of HIF-1α in the upregulation of these angiogenic factors, we blocked HIF-1α protein accumulation using digoxin, and observed a complete inhibition of endothelial cell tubule formation by conditioned medium from hypoxic UM cells. We further observed that conditioned medium from UM cells cultured in 20% O_2_ but treated with a hydroxylase inhibitor (DMOG or DFO) also promoted tubule formation by endothelial cells. These findings were corroborated using shRNA to inhibit HIF-1α expression in 92.1 UM cells (Figure 2B-2D). These results demonstrate that HIF-1α protein accumulation is necessary to promote the angiogenic phenotype of UM cells.

### HIF-1α-dependent VEGF expression contributes to the angiogenic potential of UM cells

**Figure 3:**
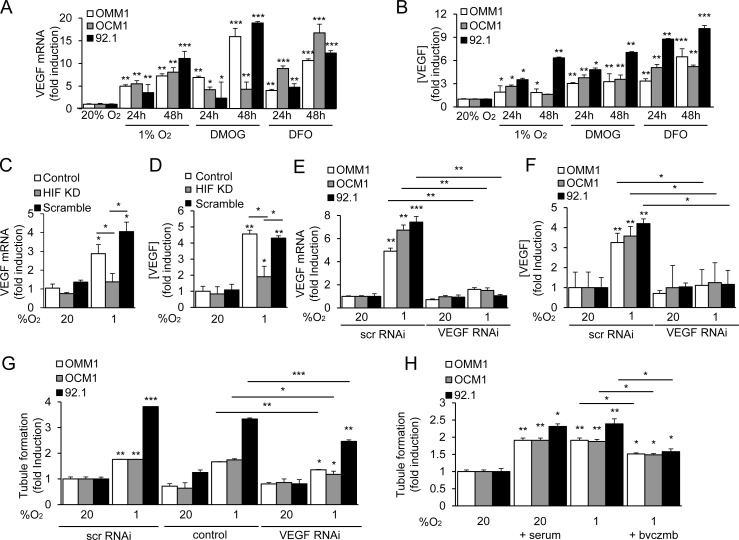
HIF-1α-dependent VEGF expression contributes to the angiogenic potential of UM cells **A.**, **B.** VEGF mRNA expression **A.** and protein secretion **B.** by UM cells exposed to hypoxia (1% O_2_) or prolyl hydroxylase inhibitor (300 μM DMOG or 100 μM DFO) in 20% O_2_ were determined. **C.**, **D.** VEGF mRNA expression **C.** and protein secretion **D.** were determined in parental 92.1 cells (Control) and in subclones expressing HIF-1α shRNA (HIF KD) or a scrambled shRNA (Scramble) that were exposed to 20% or 1% O_2_. **E.**, **F.** VEGF mRNA expression **E.** and protein secretion **F.** were determined in UM cells treated with a scrambled (scr) short interfering RNA (siRNA) or siRNA targeting VEGF. **G.** The effect on tubule formation of conditioned medium from parental cells (control), or cells transfected with scr or VEGF siRNA and exposed to 20% or 1% O_2_ was determined. **H.** Aliquots of conditioned medium from UM cells, which were cultured under serum starved conditions (1% FBS) and exposed to 20% or 1% O_2_ in the presence or absence of serum or bevacizumab (bvczmb), were tested for their effects on tubule formation. *P < 0.05; **P < 0.01; ***P < 0.001.

Vascular endothelial growth factor (VEGF) is the best studied HIF-1-regulated angiogenic factor [22]. VEGF mRNA and protein expression were increased in metastatic and primary UM cells exposed to hypoxia, DMOG, or DFO (Figure 3A and 3B). Knockdown of HIF-1α expression with shRNA significantly decreased the induction of VEGF mRNA and protein expression in 92.1 UM cells (Figure 3C and 3D; Supplementary Figure 1). These results demonstrate that VEGF is a HIF-1-regulated angiogenic factor secreted by primary and metastatic UM tumor cells.

To further interrogate the role of VEGF as a mediator of the angiogenic phenotype in UM, we utilized RNA interference (RNAi) to inhibit expression of VEGF mRNA and protein expression (Figure 3E and 3F). When endothelial cells were treated with conditioned medium from UM cells in which VEGF expression was knocked down, we observed only partial inhibition of tubule formation (Figure 3G). Using the potent VEGF-neutralizing monoclonal antibody bevacizumab, we similarly only partially inhibited tubule formation by endothelial cells treated with conditioned medium from UM cells (Figure 3H). Conversely, bevacizumab completely abolished the ability of recombinant human (rh)VEGF - at doses 5 to 10 fold higher than those measured in conditioned medium from UM cells - to promote tubule formation by treated endothelial cells (Supplementary Figure 2). Collectively, these data suggest that primary and metastatic UM cells secrete angiogenic factors in addition to VEGF.

### ANGPTL4 is a HIF-1α-regulated angiogenic factor that is expressed by UM cells

**Figure 4:**
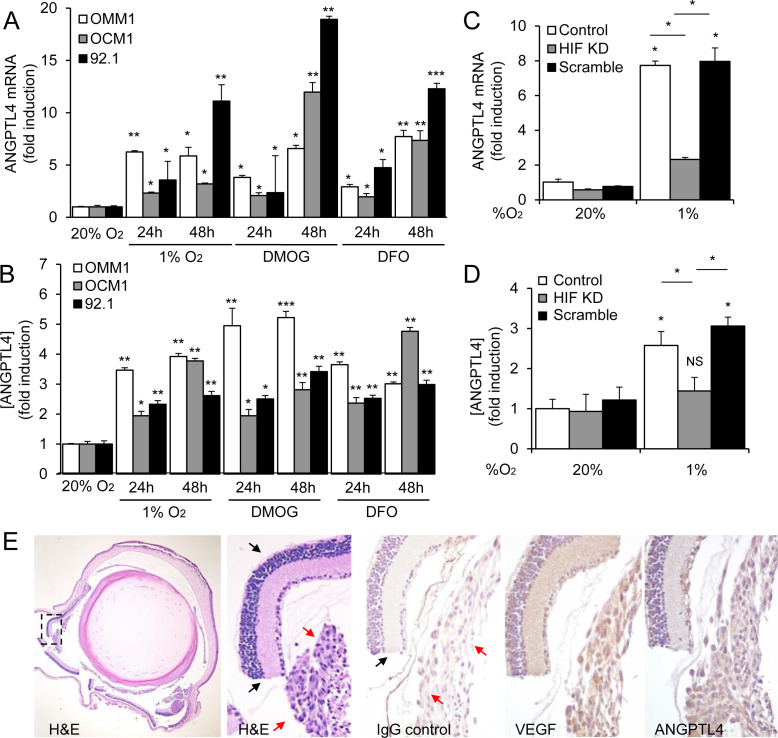
ANGPTL4 is a HIF-1-regulated angiogenic factor expressed by UM cells **A.**, **B.** ANGPTL4 mRNA expression **A.** and protein secretion **B.** in UM cell lines (OMM1, OCM1 and 92.1) exposed to hypoxia (1% O_2_), DMOG (300 μM), or DFO (100 μM) in 20% O_2_ compared to control conditions (20% O_2_) were determined. **C.**, **D.** ANGPTL4 mRNA expression **C.** and protein secretion **D.** were determined in parental 92.1 cells (Control) and in subclones expressing HIF-1α shRNA (HIF KD) or a scrambled shRNA (Scramble) that were exposed to 20% or 1% O_2_. **E.** Representative images are shown from hematoxylin and eosin (H&E) staining and immunohistochemical analysis of VEGF and ANGPTL4 expression in tumors formed following intravitreal injection of OCM1 cells into mice. Similar results were observed in 3/3 tumors analyzed. IgG was used as a negative control. Normal retina (black arrows) and UM tumor cells (red arrows) are labeled. *P < 0.05; **P < 0.01; ***P < 0.001.

Several other HIF-1-regulated secreted factors have been implicated in the promotion of angiogenesis in cancer. However, their relative contribution to angiogenesis in UM remains unclear. We examined the mRNA expression of several HIF-1-regulated angiogenic factors in the UM cells and observed upregulation of several gene products (Supplementary Figure 3). Interestingly, expression of one HIF-1-regulated gene product, Angiopoietin-like 4 (ANGPTL4), was markedly increased in all three UM cell lines. ANGPTL4 is a secreted factor that plays an important role in lipid metabolism [24], but recently has been implicated in the regulation of angiogenesis [25, 26] and metastasis [27, 28]. We therefore investigated whether ANGPTL4 participates in the promotion of angiogenesis by UM cells. ANGPTL4 mRNA and protein expression were increased when cell lines derived from metastatic or primary UM were exposed to hypoxia, DMOG, or DFO (Figure 4A and 4B; Supplementary Figure 4). Knockdown of HIF-1α expression with shRNA markedly impaired the induction of ANGPTL4 mRNA and protein expression in UM cells (Figure 4C and 4D; Supplementary Figure 5). To determine whether ANGPTL4 was expressed in UM cells *in vivo*, we performed orthotopic (intraocular) transplantation using the OCM1 cell line and observed uniform expression of ANGPTL4 in tumor cells, similar to the expression of VEGF (Figure 4E).

### ANGPTL4 promotes the angiogenic potential of UM cells

**Figure 5:**
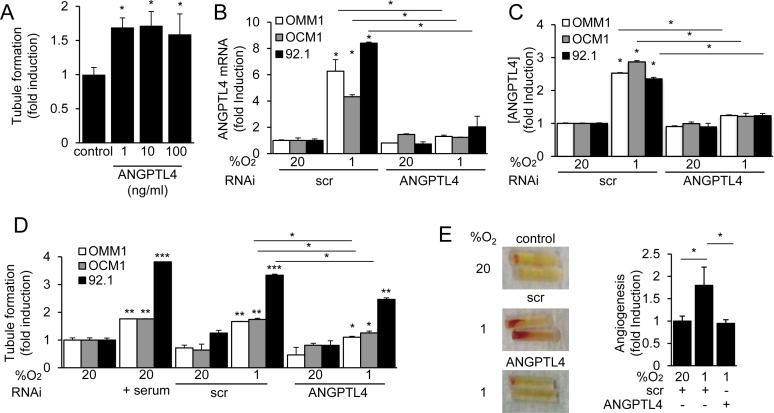
ANGPTL4 and VEGF promote the angiogenic potential of UM cells **A.** Recombinant human ANGPTL4 (1, 10 or 100 ng/mL) was tested for its effect on HMEC tubule formation. **B.**, **C.** ANGPTL4 mRNA expression **B.** and protein secretion **C.** were determined in UM cells treated with a scrambled (scr) siRNA or siRNA targeting ANGPTL4. **D.** The effect on tubule formation of conditioned medium from parental cells (control), or cells transfected with scr or ANGPTL4 siRNA and exposed to 20% or 1% O_2_, was determined. Treatment with 10% FBS was used as a positive control for the tubule formation assay. **E.** The effect of conditioned medium from cells transfected with scr or ANGPTL4 siRNA and exposed to 20% (control) or 1% O_2_ on angiogenesis *in vivo* was determined using the directed *in vivo* angiogenesis assay. Representative angioreactors (*left*) and fold induction compared to control (*right*) are shown. *P < 0.05; **P < 0.01; ***P < 0.001.

ANGPTL4 has been reported to have either pro- or anti-angiogenic, as well as either pro- or anti-metastatic effects in different tumor types [29]. Recombinant human ANGPTL4 induced tubule formation by endothelial cells (Figure 5A) at doses that were similar to the concentrations observed in conditioned medium from UM cells. To interrogate the role of ANGPTL4 in the regulation of angiogenesis by UM tumor cells, we next knocked down expression of ANGPTL4. RNAi targeting ANGPTL4 resulted in a significant inhibition of ANGPTL4 mRNA and protein expression in the three UM cells (Figure 5B and 5C). Inhibition of ANGPTL4 expression in UM tumor cells, in turn, reduced the induction by conditioned medium of endothelial cell tubule formation *in vitro* (Figure 5D) and the promotion of angiogenesis *in vivo* (Figure 5E). These results indicate that ANGPTL4 plays a pro-angiogenic role in UM.

### ANGPTL4 and VEGF are expressed and promote angiogenesis in UM tissue

**Figure 6:**
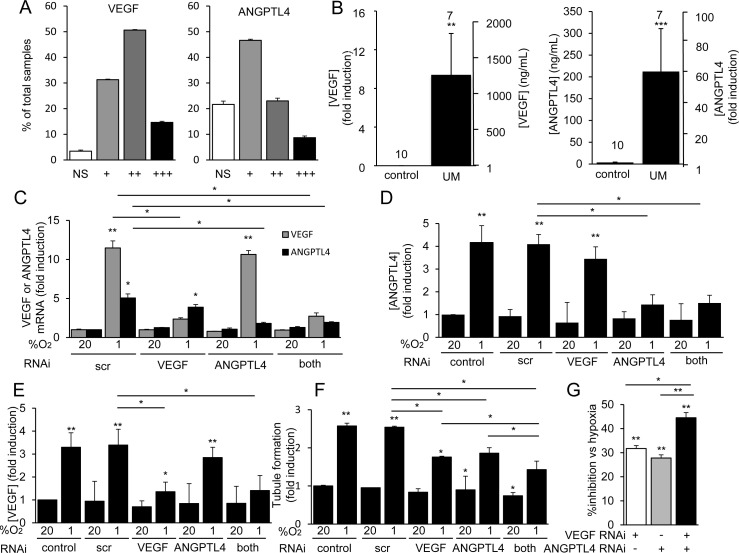
ANGPTL4 and VEGF are expressed and are angiogenic in UM tissue **A.** Core biopsies from 80 pathology-confirmed primary UMs (in quadruplicate) were used to generate a UM array. The percentage of tumor biopsies in which expression of VEGF (*left*) or ANGPTL4 (*right*) was detected by immunohistochemical analysis was determined. Expression was graded as weak (+), modest (++), or strong (+++). NS = no staining. **B.** Expression levels of VEGF (*left*) and ANGPTL4 (*right*) in the vitreous of eyes of patients with primary UM as compared to control patients without UM were determined. **C.**-**G.** VEGF and ANGPTL4 mRNA expression **C.** and protein secretion **D.**, **E.** were determined in 92.1 cells treated with a scrambled (scr) short interfering RNA (siRNA) or siRNA targeting VEGF, ANGPTL4, or both VEGF and ANGPTL4 and exposed to hypoxia (1% O_2_) as compared to control conditions (20% O_2_). **F.**, **G.** The effect on tubule formation of conditioned medium from 92.1 cells transfected with a scrambled (scr) siRNA or siRNA targeting VEGF, ANGPTL4, or both VEGF and ANGPTL4 and exposed to 20% or 1% O_2_ was determined. Results are shown as % induction compared to untreated control **F.** or % inhibition of induction compared to scr siRNA. **G.** *P < 0.05; **P < 0.01; ***P < 0.001.

To provide a quantitative analysis of VEGF and ANGPTL4 expression in primary UM, we generated a tissue array that consisted of core biopsies from 80 primary UM tumors (in quadruplicate). Immunohistochemical analysis of the array revealed that expression of VEGF was detected in tumor cells in 96% of UM biopsies (Figure 6A). ANGPTL4 expression was detected in UM tumor cells in 78% of biopsies (Figure 6A). Expression of either VEGF or ANGPTL4 was detected in 99% of biopsies.

Next, we obtained vitreous samples from UM patients with primary tumors who underwent enucleation and detected a marked increase in ANGPTL4 in the vitreous of eyes with UM compared to vitreous biopsies from control patients without UM (Figure 6B; Supplemental Figure 6). Vitreous samples from 5 of 7 UM patients had elevated levels of ANGPLT4. There is a striking correlation between the levels of ANGPTL4 and VEGF (Supplemental Figure 6), which is consistent with their coordinate regulation by HIF-1. There is also a strong correlation between the levels of ANGPTL4 and VEGF in UM patients; the levels of ANGPTL4 and VEGF co-increased in 4/7 UM patients (Supplemental Figure 7).

To explore whether combined therapies targeting both VEGF and ANGPTL4 could be an effective approach to inhibit angiogenesis in UM, we knocked down expression of VEGF, ANGPTL4, or both. RNAi targeting either VEGF or ANGPTL4 in 92.1 cells inhibited VEGF or ANGPTL4 mRNA and protein expression, respectively, and did not impact the expression of each other (Figure 6C-6E). Combined RNAi knockdown blocked the mRNA and protein expression of both secreted factors and had an additive effect on the inhibition of tubule formation by endothelial cells treated with conditioned medium from the 92.1 UM cells (Figure 6F and 6G). Collectively, these data demonstrate that VEGF and ANGPTL4 independently contribute to the angiogenic phenotype in UM.

## DISCUSSION

Current treatment options for local UM disease - including eye-sparing approaches (e.g. radioactive plaque therapy or laser therapy) - often lead to profound vision loss [30]. Moreover, despite the growing use of gene expression profiling that may identify which patients are likely - or unlikely - to develop metastatic disease [31], there is no effective adjuvant treatment available to prevent or treat metastases in patients who receive a diagnosis of UM. Ultimately, development of novel gene product-targeted therapeutic options that would avoid tissue destruction for local disease, yet effectively treat or prevent metastases, is essential.

In this regard, the formation of new blood vessels constitutes a prerequisite for the growth of solid tumors [5]. Expression of many oncogenes promotes tumor neovascularization by inducing the release of angiogenic factors such as VEGF. *In vitro* studies have revealed that UM cells express VEGF under non-hypoxic culture conditions, and that expression further increases under hypoxic conditions [32, 33]. Recent studies have confirmed that patients with UM have increased vitreous levels of VEGF [34, 35] and our results corroborate these studies. Expression of VEGF within primary UM tumors has been less clear, ranging from 26% in some studies to 94% in others [36, 37]. Using a UM tumor array, we demonstrate here that VEGF expression is detected in 96% of UM tumors samples, with moderate to high levels detected in approximately two-thirds of tumors.

There are conflicting reports regarding correlations between expression levels of VEGF and tumor size, vascularization, or metastasis. Nonetheless, the availability of humanized monoclonal antibodies targeting VEGF, which were introduced to treat other ocular neovascular diseases, has made anti-VEGF therapy an attractive approach as an adjuvant treatment for UM, and results from a recent clinical trial using intravitreal injections of bevacizumab to reduce the size of local UM are pending (ClinicalTrials.gov identifier: NCT00596362). Our studies using RNAi and neutralizing antibody against VEGF lend further support for the possible benefit of bevacizumab as an adjuvant therapy for UM. However, we observe only partial inhibition of the angiogenic potential of UM tumor cells by targeting VEGF alone, suggesting that additional secreted angiogenic factors play a role in UM pathogenesis.

In addition to driving primary tumor growth and progression, angiogenesis also plays a pivotal role in tumor invasiveness and metastasis. This is particularly true for hematogenous metastasis, and the vascular system is critical for metastasis in UM. In a pre-clinical study, bevacizumab has been reported to suppress hepatic micrometastasis of UM cells [38]. Consequently, there is some hope that therapies targeting VEGF may also prevent (or slow) metastatic spread. However, studies from other metastatic tumors have demonstrated that the benefit in patients treated with anti-VEGF therapy has been limited to only modest (several months) or no improvement in overall survival [39]. Moreover, preclinical studies suggest that anti-VEGF therapies may reduce primary tumor growth but paradoxically promote tumor invasiveness and metastasis by increasing intratumoral hypoxia and HIF-1 activity [40].

In the present study, we demonstrate that ANGPTL4, another HIF-1-regulated secreted protein, is also expressed by UM cells. Angiopoietins are a family of secreted factors that are critical for vascular development [41]. Angiopoietin-1 (ANGPT-1) promotes vessel maturation, whereas angiopoietin-2 (ANGPT-2) antagonizes its effect on vessel stabilization. Angiopoietin-like proteins (ANGPTLs) are structurally similar to the angiopoietins. However, ANGPTL proteins do not bind to the ANGPT receptors, TIE1 and TIE2, and exhibit a plethora of functional roles, including the regulation of lipid and glucose metabolism, inflammation, and cancer [42, 43]. Importantly, recent studies support a role for ANGPTL4 as a regulator of tumor angiogenesis. Although initially considered to be anti-angiogenic [44-47], subsequent work has demonstrated that ANGPTL4 promotes the angiogenic and exudative phenotypes that are characteristic of the unique vascular tumor, Kaposi's sarcoma [25]. More recently, ANGPTL4 has been shown to promote vascular permeability and pathological angiogenesis in ischemic retinal disease [26, 48].

We demonstrate that ANGPTL4 participates with VEGF in the promotion of angiogenesis in UM. Inhibition of ANGPTL4 expression by UM tumor cells reduced the induction of endothelial cell tubule formation *in vitro* and the promotion of angiogenesis *in vivo*. Using a tumor array, we demonstrate expression of ANGPTL4 in almost 80% of UM tumors, with expression of either VEGF or ANGPTL4 in 99% of primary UM tumors. Interestingly, vitreous samples from 5 of 7 patients with UM patients had elevated levels of ANGPLT4. These patients are likely to have an incomplete response to anti-VEGF therapy alone.

In this regard, we demonstrate that inhibiting expression of both VEGF and ANGPTL4 by UM tumor cells was more effective in preventing secretion of angiogenic factors as compared to inhibiting expression of either angiogenic protein alone. This suggests that therapies targeting ANGPTL4 in combination with current anti-VEGF approaches may be a more effective anti-angiogenesis approach for the treatment of UM. Of note, inhibition of both ANGPTL4 and VEGF expression by RNAi was not sufficient to completely abolish the angiogenic potential of UM tumor cells *in vitro*. This may be a consequence of the failure of RNAi to completely block ANGPTL4 and/or VEGF expression. However, we further demonstrate that pharmacological inhibition of HIF-1 effectively blocked the angiogenic potential of UM cells *in vitro*. Collectively, these data suggest that additional HIF-regulated secreted factors may participate in the promotion of the angiogenic phenotype in UM.

In addition to its pro-angiogenic function, ANGPTL4 has also been shown to promote vascular permeability*, via* disruption of the integrity of endothelial adherens junctions and tight junctions [25]. This ANGPTL4-induced junction disassembly is dependent on a rapid activation of the RhoA/ROCK signaling pathway and the disruption of VE-cadherin and claudin-5 clusters. Destabilization of the endothelial barrier is required for trans-endothelial passage of cancer cells (extravasation) and consequent tissue colonization in the metastatic process [25, 28]. In breast cancer, extravasation of hypoxic tumor cells into the lung is dependent on HIF-1-regulated expression of ANGPTL4 [27]. Further studies are needed to determine whether inhibiting ANGPTL4, by preventing its promotion of endothelial cell barrier disruption, may also be an effective approach for the prevention of UM cell extravasation and metastatic spread. Although a role for ANGPTL4 in promoting tumor cell intravasation has not been explored, ANGPTL4 also plays an important role in promoting vascular permeability of retinal vessels in ischemic retinal disease [48]. Similarly, ANGPTL4 may also promote the transition from subclinical micro-metastasis to symptomatic macro-metastasis by facilitating metastatic tumor growth. Collectively, these observations support a possible role for ANGPTL4 in the promotion of metastasis in UM and provide a foundation for future studies to determine whether combined inhibition of both ANGPTL4 and VEGF could simultaneously target tumor-induced angiogenesis and metastasis, and thereby provide more effective therapies for patients with primary and metastatic UM.

## MATERIALS AND METHODS

### Constructs and reagents

Recombinant human ANGPTL4, and VEGF, as well as ANGPTL4 (DuoSet) and VEGF (DuoSet) ELISA kits were purchased from R&D Systems. Predesigned control (scrambled), ANGPTL4 and VEGF small interfering RNAs (siRNAs) were obtained from Santa Cruz. Lipofectamine RNAiMAX transfection reagent was obtained from Life Technologies. Digoxin and DFO were obtained from Sigma. DMOG was obtained from Cayman Pharmaceuticals.

### Cell culture

92.1, OCM1 and OMM1 UM cell lines were kindly provided by Dr. J. Niederkorn (2007, UT Southwestern Medical Center, Dallas, TX) and cultured with RPMI-1640 (Invitrogen) containing L-glutamine (Invitrogen), HEPES (Gibco), sodium pyruvate (Gibco) and non-essential amino acids (Gibco) supplemented with 10% FBS (Invitrogen) and 1% penicillin/streptomycin (Cellgro). Lentiviral constructs containing PLKO.1 transfer vector with short hairpin RNA (shRNA) targeting HIF-1α mRNA, whose target sequences were previously shown [15], were purchased from Thermo Fisher Scientific (Waltham, MA). Lentiviral particles were prepared using HEK293T cells as previously described (15). Puromycin (5 μg/mL) was used to select the cells expressing the transfer vector. Scrambled shRNA was used as control. Immortalized human dermal microvascular endothelial cells (HMEC) were obtained from the Center for Disease Control and Prevention (CDC) and cultured with DMEM containing 4.5 g/l glucose with 10% FBS and 1% penicillin/streptomycin. Before treatment, the growth medium was replaced with medium containing 1% FBS. UM cells were exposed to 1% O_2_ using an Oxygen Controller Glove Box (Coy Laboratory Products Inc.), equilibrated with a gas mixture containing 1% O_2_, 5% CO_2_, and 94% N_2_ at 37°C. UM cell lines were tested (OMM1 April, 2015, 92.1 and OCM1, 2010) and their identity authenticated at the Johns Hopkins Molecular Core Laboratory through short tandem repeat (STR) analysis. Authentication was not performed on HEK293T or HMECs.

### siRNA transfection

Cells were seeded and grown to 60-80% confluence prior to transfection. Lipofectamine RNAiMAX reagent was diluted in Opti-MEM medium. 30 pmol of siRNA from stock of 10 μM was diluted in Opti-MEM medium. Diluted siRNA was added to diluted Lipofectamine RNAiMAX reagent (1:1 ratio) and incubated for 5 minutes at room temperature. siRNA-lipid complex was added to cells and incubated at 37°C for 24 hours. The medium was then washed out and cells were ready for experiments.

### Western blot assays

Cells in culture dishes were washed with PBS and lysed using RIPA buffer (Sigma) with 10% protease inhibitor cocktail (Sigma). Cell lysates were then solubilized in LDS-sample buffer (Life Technologies) and incubated for 5 minutes at 95°C. Lysates were subjected to 4-15% gradient SDS/PAGE (Invitrogen). After blocking the membrane with 5% milk (Bio-Rad), the membrane was then incubated with mouse anti-HIF-1α (BD, 610959) or rabbit anti-HIF-1α (Abcam, 2185) or with mouse anti-GAPDH monoclonal antibody (Fitzgerald) overnight at 4°C. After washing, the membrane was incubated with (HRP)-conjugated anti-mouse or anti-rabbit IgG (Cell Signaling) for 1 hour and then visualized with ECL Super Signal West Femto (Thermo). Western blot scans are representative of at least three independent experiments.

### Quantitative real-time RT-PCR

mRNA was isolated from cultured cells or isolated retinas with RNeasy Mini Kit (Qiagen), and cDNA was prepared with MuLV Reverse Transcriptase (Applied Biosystems). Quantitative real-time PCR was performed with Power SYBR Green PCR Master Mix (Applied Biosystems) and MyiQ Real-Time PCR Detection System (Bio-Rad). β-actin was used for normalization of human cell lines. Primers for qPCR include: VEGF, forward - GGGCAGAATCATCACGAAGT and reverse - TGGTGATGTTGGACTCCTCA; ANGPTL4, forward - GGACACGGCCTATAGCCTG and reverse - CTCTTGGCGCAGTTCTTGTC; β-actin, forward - CTCTTCCAGCCTTCCTTCCT and reverse - AGCACTGTGTTGGCGTACAG.

### ELISA

Vitreous diluted 1:1 and 1:10 and conditioned medium diluted 1:1 were analyzed for ANGPTL4 and VEGF with ELISAs performed according to the manufacturer's protocols (R&D Systems).

### Endothelial cell tubule formation assay

Endothelial cell tubule formation assay was performed using growth factor-reduced Matrigel (BD Biosciences; 356231). 60-80 μl of Matrigel was added into a pre-chilled 96-well plate and placed in a 37°C CO_2_ incubator for 30 minutes. HMECs were then counted and plated at 2 × 10^4^ cells/well on the Matrigel in a 96-well plate. Eighteen hours later, images were captured and analyzed using ImageJ software. Tubule formation assay with conditioned medium from UM cells was performed with an addition of 100 μl/well of conditioned medium to the cell suspension prior to adding into the Matrigel-coated wells. VEGF neutralization was performed using 100 μg/ml of bevacizumab (JHU Pharmacy).

### Directed *in vivo* angiogenesis assay

The assay was performed according to the manufacturer's protocol (Trevigen). Briefly, four silicone cylinders (angioreactors) filled with matrigel and conditioned medium from UM cells cultured under different culture conditions were subcutaneously implanted into the dorsal flank of each nu/nu mice (Charles River Laboratories). At day 14 the animals were euthanized by cervical dislocation and the angioreactors were collected and analyzed.

### Hematoxylin and eosin (H&E) staining

After treating cryosections and paraffin-embedded sections in absolute methanol for 5 min at 20°C and allowing them to air dry, sections were stained with Harris' hematoxylin for 20 sec. After washing in distilled water, the sections were blued in lithium carbonate, rinsed in distilled water, and then stained in 0.5% alcoholic eosin (Polysciences). After dehydration to xylene, the sections were coverslipped with Permount (Fisher Scientific).

### Immunofluorescence

Immunofluorescence followed by confocal microscopy was used in cryopreserved uveal melanoma tissue. The primary antibody used: HIF-1α (Abcam) was performed as previously described [21]. After washing, the sections were labeled with secondary antibody goat anti-rabbit Alexa F 488 associated with DAPI (Invitrogen). Images were captured using the LSM 710 Meta confocal microscope (Carl Zeiss).

### Immunohistochemistry

Streptavidin alkaline phosphatase (APase) immunohistochemistry was performed on cryopreserved tissue sections using a nitroblue tetrazolium (NBT) development system as previously described [21]. An ABC system (Dako) was performed in paraffin-embedded mouse tissue as previously described [21]. Primary antibodies used include: HIF-1α (ABCAM), ANGPTL-4 (ABCAM), VEGF (Santa Cruz) after dilution in TBS with 1% bovine serum albumen (BSA). All immunohistochemical reagents, including antibodies, were identical for all specimens.

### Mice

All studies involving mice were approved by the Johns Hopkins University Animal Care and Use Committee and were performed in accordance with the NIH Guide for the Care and Use of Laboratory Animals. For orthotopic (intraocular) tumor cell transplantation, female athymic 5-week-old nude mice (NU/J, Jackson Laboratory) were deeply anesthetized with ketamine hydrochloride (Sigma-Aldrich) and tumor cell transplantation was performed using 5×10^4^ OCM1 cells injected into the vitreous of the right eye using a 33-gauge needle. Mice were euthanized after three weeks, and the eyes were sectioned, stained with H&E, and analyzed by light microscopy for the presence of tumor by an experienced pathologist (CGE).

### Tumor arrays

Core biopsies (0.6 mm in diameter) of 80 UMs (4 cores each) were arrayed. Immunohistochemical staining was graded in a blinded fashion by two independent investigators (MR and AS) as no staining (NS), weak staining (+), modest staining (++), or strong staining (+++).

### Patient samples

Institutional Review Board approval from the Johns Hopkins University School of Medicine was obtained for all patient samples used in this study. Eyes were collected from patients with UM requiring enucleation. Vitreous was extracted from enucleated eyes and stored at −80°C. Frozen vitreous samples were thawed and centrifuged at 16,000 x g for 5 minutes at 4°C prior to analysis.

### Statistical analysis

Results from clinical samples are shown as mean ± SEM. Statistical differences between groups were determined by the Mann-Whitney U test. Results from cell culture and animal models are shown as mean ± SEM from at least three independent experiments. Statistical differences between groups were determined by Student's t-test or one-way ANOVA as indicated. Statistical analysis was performed using Microsoft Office and Prism 6.0 software (GraphPad).

## SUPPLEMENTARY MATERIALS AND FIGURES


