# Correction: Hypoxia-inducible factor 1 upregulation of both VEGF and ANGPTL4 is required to promote the angiogenic phenotype in uveal melanoma

**DOI:** 10.18632/oncotarget.27780

**Published:** 2021-03-02

**Authors:** Ke Hu, Savalan Babapoor-Farrokhran, Murilo Rodrigues, Monika Deshpande, Brooks Puchner, Fabiana Kashiwabuchi, Syed Junaid Hassan, Laura Asnaghi, James T. Handa, Shannath Merbs, Charles G. Eberhart, Gregg L. Semenza, Silvia Montaner, Akrit Sodhi

**Affiliations:** ^1^ Wilmer Eye Institute, Johns Hopkins School of Medicine, Baltimore, MD, USA; ^2^ The First Affiliated Hospital of Chongqing Medical University, Chongqing, China; ^3^ Department of Pathology, Johns Hopkins University, School of Medicine, Baltimore, MD, USA; ^4^ Departments of Pediatrics, Medicine, Oncology, Radiation Oncology, Biological Chemistry, and Genetic Medicine, Johns Hopkins University School of Medicine, Baltimore, MD, USA; ^5^ Department of Oncology and Diagnostic Sciences, Greenebaum Cancer Center, University of Maryland, Baltimore, MD, USA

**This article has been corrected:** In Figure 1, panel D, two of the HIF-1 alpha images were mislabeled. Specifically, the HIF-1 alpha image of OCM1 cells exposed to 1% oxygen was labeled as “DFO” and the HIF-1 alpha image of OCM1 cells exposed to DFO was labeled as “1% oxygen.” The corrected Figure 1 is shown below. The authors declare that these corrections do not change the results or conclusions of this paper.

Original article: Oncotarget. 2016; 7:7816–7828. 7816-7828. https://doi.org/10.18632/oncotarget.6868

**Figure 1:**
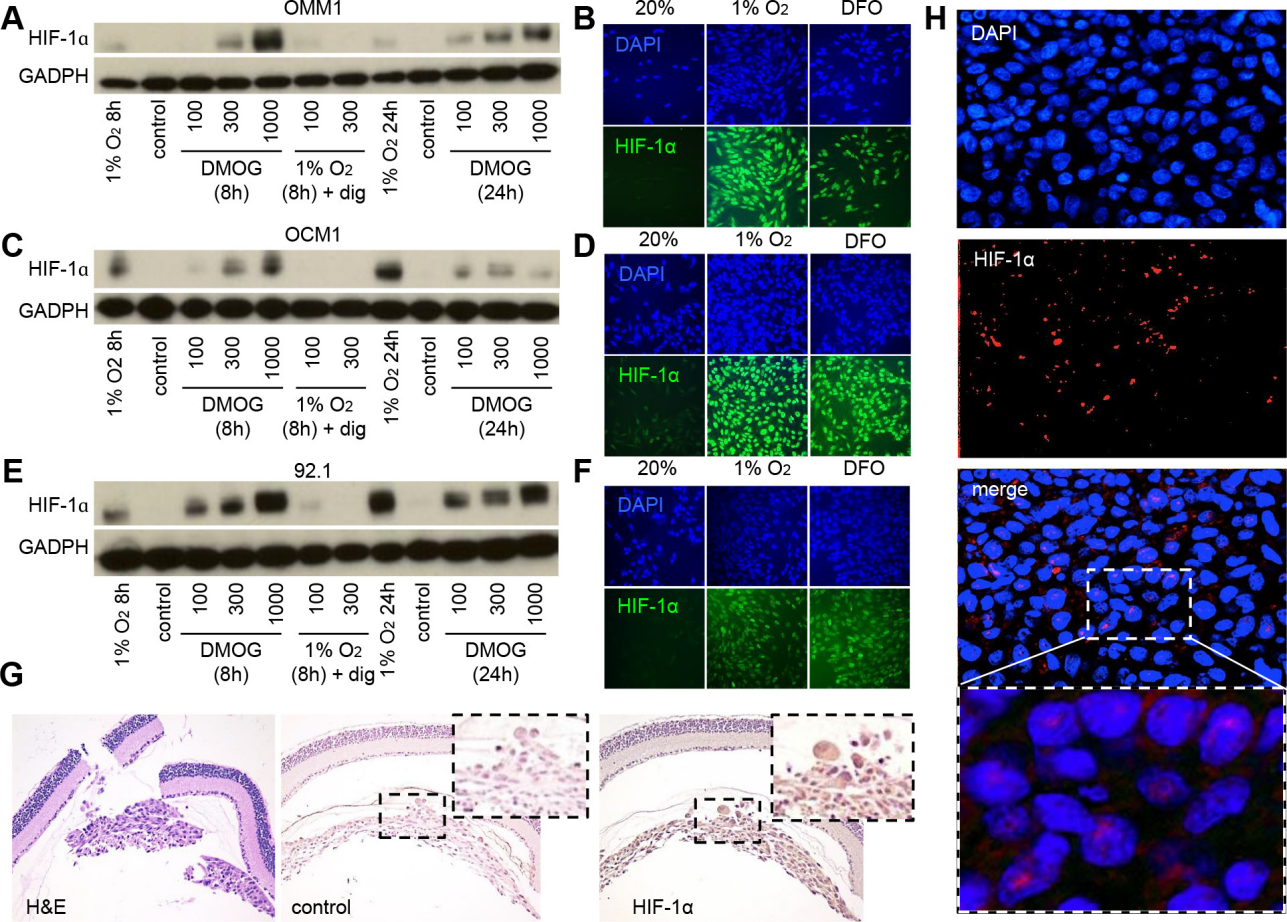
HIF-1α expression is increased in UM cells and in UM patient biopsies. (**A**, **C**, **E**) Immunoblot assays were performed to determine HIF-1α protein levels in UM cell lines (OMM1, OCM1 and 92.1) following exposure to DMOG (300 μM), hypoxia (1% O_2_) or hypoxia and digoxin (dig; 100-300 nM) for 8 or 24 hours and compared to control conditions (20% O2). (**B**, **D**, **F**) Representative images are shown from immunofluorescence analysis of HIF-1α in UM cell lines following exposure to hypoxia (1% O2 for 8 or 24 hours)or DFO (100 μM for 8 or 24 hours). (**G**) Representative images are shown from immunohistochemical analysis of HIF-1α expression in tumors formed following intravitreal injection of OCM1 cells into mice. Similar results were observed in 3/3 tumors analyzed. (**H**) Representative images are shown from immunofluorescence analysis of HIF-1α protein accumulation and nuclear localization in a human UM tumor biopsy. Similar results were observed in 6/6 UM biopsies examined.

