# Supplementary material for: Hypoxia-inducible factor 1 upregulation of both VEGF and ANGPTL4 is required to promote the angiogenic phenotype in uveal melanoma
Source: Oncotarget. 2016 Jan 9;7(7):7816–28. doi: 10.18632/oncotarget.6868 (PMC4884956; doi:10.18632/oncotarget.6868)
Supplement: Supplementary file 1 [file oncotarget-07-7816-s001.pdf]

Hypoxia-inducible factor 1 upregulation of both VEGF and ANGPTL4 is required to promote the angiogenic phenotype in uveal melanoma

Supplementary Materials and Methods

Quantitative real-time RT-PCR

mRNA was isolated from cultured cells with RNeasy Mini Kit (Qiagen), and cDNA was prepared with MuLV Reverse Transcriptase (Applied Biosystems). Quantitative real-time PCR was performed with Power SYBR Green PCR Master Mix (Applied Biosystems) and MyiQ Real-Time PCR Detection System (Bio-Rad). β-actin was used for normalization of human cell lines. Primers for qPCR include: PIGF, forward - GTTCAGCCCATCCTGTGTCT and reverse – TTCATCTTCTCCCGCAGAG; bFGF, forward – CCACTTCAAGGACCCCAAG and reverse - TGAGGGTCGCTCTTCTCC; PDGF, forward – TTCCCTGACCATTGCTGA and reverse – AGGAAGTTGGCGTTGGTG ; PEDF, forward -TGAGAAGAAGCTGCGCATAA and reverse – ACCGAGAAGGAGAATGCTGA; EPO, forward -CTCCGAACAATCACTGCT and reverse – GGTCATCTGTCCCCTGTCCT; ANGPTL4, forward –GGACACGGCCTATAGCCTG and reverse –CTCTTGGCGCAGTTCTTGTC; VEGF, forward –GGGCAGAATCATCACGAAGT and reverse –TGGTGATGTTGGACTCCTCA; BMP7, forward – TCCGGTTTGATCTTTCCAAG and reverse - ATCCGGAACGTCTCATTGTC; β-actin, forward – CTCTTCCAGCCTTCCTTCCT and reverse – AGCACTGTGTTGGCGTACAG.

	[VEGF] pg/ml	
	20% O_2	1% O_2
Control	50	227
HIF KD	24	95
Scramble	54	214

Supplemental Figure 1.
Concentration of VEGF (pg/mL) in conditioned medium from parental 92.1 cells (Control) and subclones stably expressing either a short hairpin RNA (shRNA) targeting HIF-1α (HIF KD) or a scrambled control (scr) shRNA and exposed to 1% or 20% O_2.

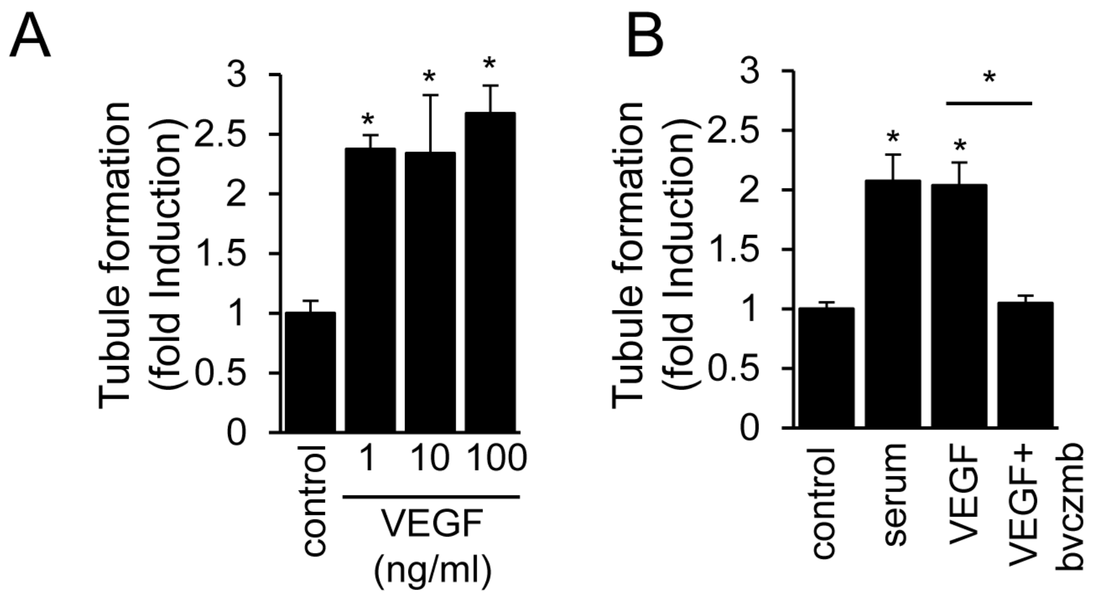

Supplemental Figure 2.
(**A**) Recombinant human VEGF (1, 10 or 100 ng/mL) was tested for its effect on HMEC tubule formation. (**B**) The effect of treatment with a saturating dose of the VEGF neurtralizing antibody, bevacizumab (bvczmb; 100 μg/ml) to competitively inhibit the ability of rhVEGF to promote the formation of tubules by treated HMECs, was determined. * $P < 0.05$.

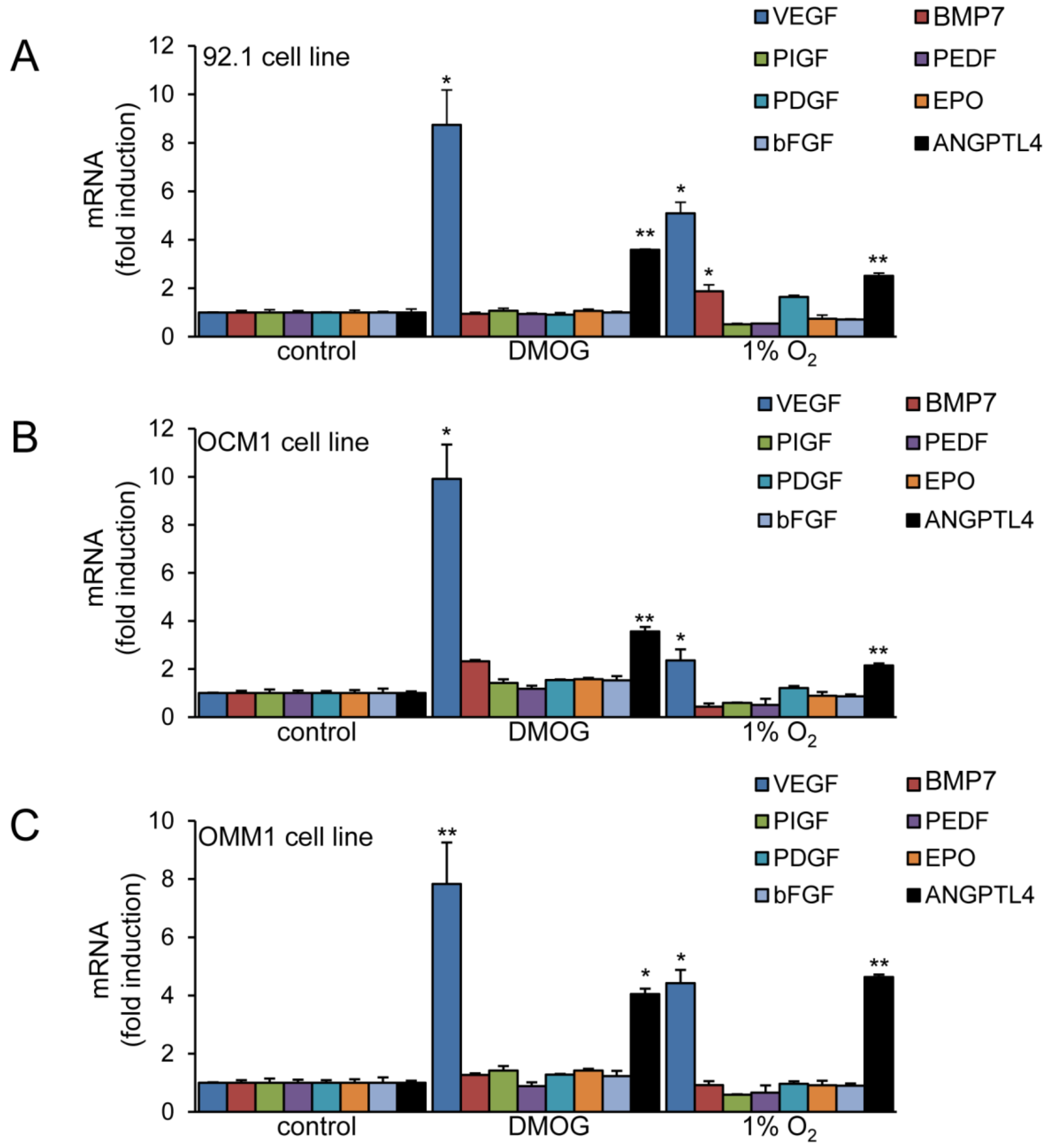

Supplemental Figure 3.
(**A** – **C**) mRNA expression of HIF-1-regulated angiogenic gene products in 92.1 (**A**), OCM1 (**B**), and OMM1 (**C**) cell lines exposed to 1% O_2 or DMOG (300 μM), for 24 hours was determined.

	rhANGPTL4 (ng/ml)						
	20% O_2	1% O_2		DMOG		DFO	
		24h	48h	24h	48h	24h	48h
OMM1	1.0	3.5	5.0	3.7	3.9	5.2	3.0
OCM1	1.9	3.6	3.6	4.4	7.0	5.3	8.9
92.1	2.1	5.0	5.3	5.4	5.5	7.3	6.4

Supplemental Figure 4.
Concentration of ANGPTL4 (ng/mL) in conditioned medium from 3 UM cell lines (OMM1, OCM1 and 92.1) exposed to 1% or 20% O_2, DMOG (300 μM), or DFO (100 μM) for 24 or 48 hours was determined.

	ANGPTL4 (ng/ml)	
	20% O_2	1% O_2
Control	2.7	7.1
HIF KD	2.5	4.0
Scramble	3.3	8.4

Supplemental Figure 5.
Concentration of ANGPTL4 (ng/mL) in conditioned medium from parental 92.1 cells (Control) and subclones stably expressing either a short hairpin RNA (shRNA) targeting HIF-1α (HIF KD) or a scrambled control (scr) shRNA and exposed to 1% or 20% O_2.

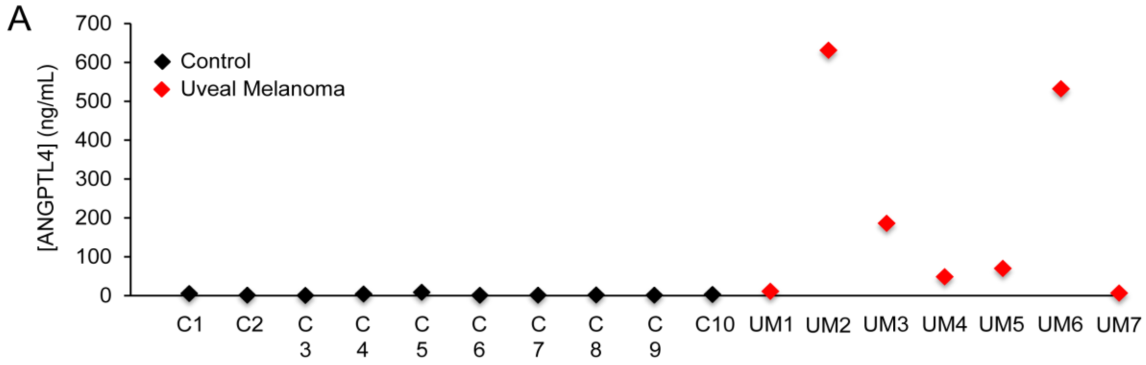

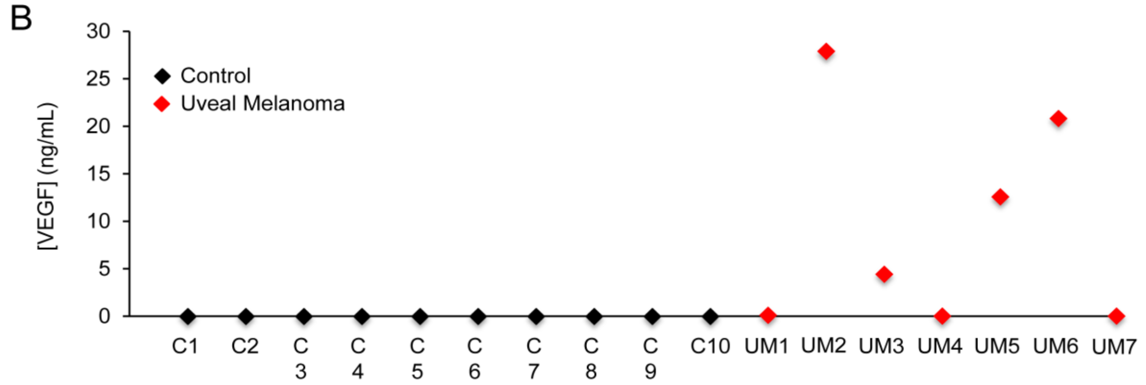

Supplemental Figure 6.
(**A,B**) Scatter plot demonstrating the expression of ANGPTL4 **(A)** and VEGF **(B)** in the vitreous of 7 UM patients (red diamonds) compared to 10 control patients without UM (black diamonds).

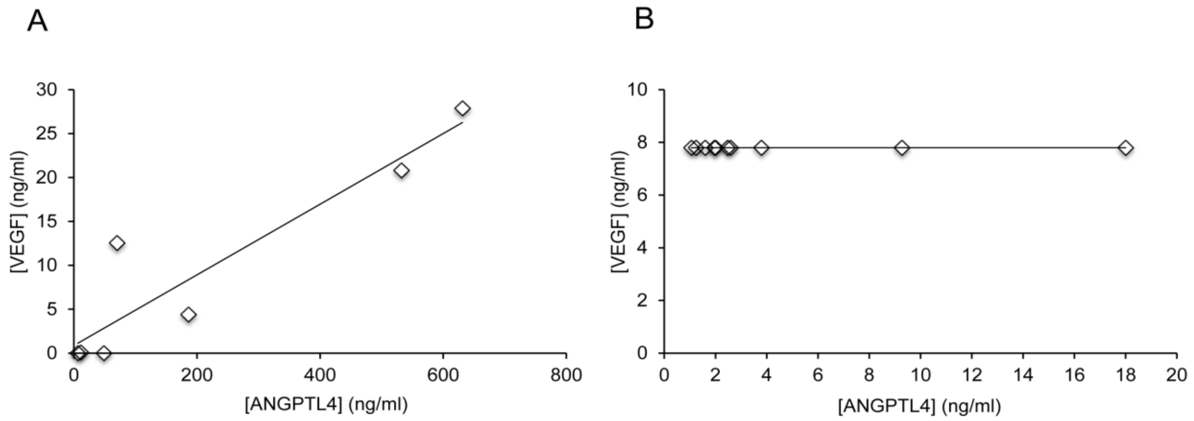

Supplemental Figure 7.
(**A**,**B**) Scatter plot demonstrating the correlation between [VEGF] and [ANGPTL4] in the vitreous of 7 UM patients (**A**) or 10 control patients without UM (**B**). Pearson's correlation.
